# Hypoglycemic potential of selenium nanoparticles capped with polyvinyl-pyrrolidone in streptozotocin-induced experimental diabetes in rats

**DOI:** 10.1016/j.heliyon.2020.e04045

**Published:** 2020-05-30

**Authors:** Ola M. El-Borady, Mohamed S. Othman, Heba H. Atallah, Ahmed E. Abdel Moneim

**Affiliations:** a Institute for Nanoscience and Nanotechnology, Kafrelsheikh University, Kafrelsheikh, Egypt; b Faculty of Preparatory Year, University of Ha'il, Hail, KSA, Saudi Arabia; c October University for Modern Science and Arts (MSA), Giza, Egypt; d Zoology and Entomology Department, Faculty of Science, Helwan University, Cairo, Egypt

**Keywords:** Biochemistry, Diabetes, Selenium, Nanoparticles, Polyvinyl-pyrrolidone, Hyperglycemia, Oxidative stress, Hypoglycemic, Streptozotocin, Materials science, Chemistry, Biological sciences, Veterinary medicine, Health sciences

## Abstract

This study was aimed to evaluate the efficacy of synthesized selenium nanoparticles (SeNPs) capped with glucose and polyvinyl-pyrrolidone (PVP) on the hyperglycemia and prooxidants/antioxidants imbalance present in model streptozotocin (STZ)-induced diabetic rats. SeNPs were synthesized and characterized. Twenty-four albino male rats were grouped into four different groups. After the rats were induced to have type 2 diabetes by STZ, the SeNPs-treated groups received a dose of 0.5 mg/ml of SeNPs for seven days. Plasma glucose and insulin levels, pancreatic insulin expression, the levels of lipid peroxidation (LPO), nitric oxide (NO), glutathione peroxidase (GPx) and glutathione (GSH) were evaluated. TEM images revealed the formation of semispherical particles with average size between 40 and 50 nm. SeNPs administration successfully reduced the hyperglycemia, raised the levels of insulin in both the pancreas and the plasma and restored the damaged pancreatic tissue. SeNPs also showed enhancement of the elimination of the diabetes-induced oxidative stress injuries by decreasing the pancreatic LPO and NO levels. Furthermore, the activities of the antioxidant enzyme GPx and GSH levels of the diabetic rats were increased. In conclusion, SeNPs capped with PVP could be used in the future as an agent that could manage Diabetes mellitus.

## 1 Introduction

Diabetes mellitus (DM) is a common metabolic endocrine disorder that remains to be a concerning epidemic in the last century and is one of the main leading causes of worldwide death (Glovaci et al., 2019). It has been estimated that in 2017, approximately 425 million adults were living with diabetes. The number of people affected are expected to increase to 629 million by 2045 (Federation, 2017). Resistance of cells to insulin or impair in insulin synthesis is the underlying causes of diabetes. Consequently, fat, carbohydrate and protein metabolism are severely disturbed (Othman et al., 2019). The deficiency or absence of insulin causes hyperglycemia; hence, an increase in the level of free radicals is noticed due to the oxidation of the increased glucose (Amin et al., 2020). Oxidative stress is thereby directly correlated with diabetes and the activity of the system specialized for antioxidant defense of the body is weakened due to hyperglycemia (AlAmri et al., 2020).

Selenium (Se) is a necessary micronutrient in our diet and it could down-regulate diabetic symptoms (Pillai et al., 2012). According to Hwang et al. (2007), Se acts like insulin in streptozotocin (STZ)-induced diabetic mice, in which it can regulate the action of several enzymes that are involved in gluconeogenesis and glycolysis and it can facilitate the transportation of glucose to cells. However, elevated Se blood levels could result in toxicity. On the other hand, SeNPs are more biocompatible with no or low toxicity when compared to selenomethionine or selenite (Deng et al., 2019). Nanotechnology is currently one of the leading scientific fields in cutting edge breakthroughs (AlBasher et al., 2019). Its interdisciplinary nature serves different fields by allowing engineering on the molecular and atomic scale to be possible (Khurana et al., 2019). Therefore, this study was aimed to fabricate SeNPs, characterization and then evaluation of its effects on STZ-induced diabetic rats.

## 2 Materials and methods

### 2.1 Synthesis of selenium nanoparticles (SeNPs)

An amount of 0.25 M of sodium seleno-sulphate was prepared according to Ingole et al. (2010), in which 20 g of sodium sulphite was mixed with 2 g of Se powder (Sigma-Aldrich) in 100 ml of distilled water for 7 h at 80 °C and then it was diluted to 0.1 M. SeNPs were synthesized by the reduction of aqueous sodium seleno-sulphate solution in which 20 ml 10^−1^ M of sodium seleno-sulphate was well mixed with 5 ml 4% glucose solution followed 0.5 g of PVP (Sigma-Aldrich) which acted as a capping agent and the solution was mixed with 30 ml of water. It was refluxed for 6 h at 70 °C. The concentration of SeNPs was calculated using the following equation: Concentration of SeNPs = Molarity of the solution x atomic weight of selenium = 0.1 M x 78.96 = 7.89 mg/ml.

### 2.2 Characterization of the SeNPs

#### 2.2.1 Absorption spectra

The absorption spectrum of the resulting solution was scanned on the visible and ultra-violet range (200–1000 nm) using the same solvent as blank. One centimeter of quartz cells was used to record the absorption spectra in a double beam spectrophotometer, model Perkin-Elemer Lambda 40 B.

#### 2.2.2 Transmission electron microscope (TEM) imaging

SeNPs were characterized using the TEM; JOEL JEM-2100 (Nanotech Company, Egypt), microscope with an accelerating voltage of 200 kV, attached to Gatan Digital Camera, Model Erlangshen ES500.

### 2.3 Animals groupings and induction of diabetes mellitus

All demonstrated experimental protocols in the current research applied on animals, were approved by the Committee of Research Ethics for Laboratory Animal Care, Department of Zoology and Entomology, Faculty of Science, Helwan University (approval no, HU2016/Z/05), following the National Institutes of Health (NIH) Guidelines applied for the use and care of Laboratory Animals, 8^th^ edition (NIH Publication no. 85–23, revised 1985). Twenty-four male albino rats (180–220 g) were delivered from the Research Institute of Ophthalmology, Animal House Department, El Giza, Egypt. Six rats were housed per cage while the environment in laboratory was adjusted at temperature = 25 ± 1 °C and a relative humidity = 50 ± 2% and animals received the water and food as necessary. After one week of acclimatization, twelve mice were used as controls; they were again randomly divided into two subgroups: the control group and the synthesized SeNPs-treated normal group. After 24 h of fasting, the other twelve rats were intraperitoneally injected with STZ according to Aboonabi et al. (2014) (45 mg/kg body weight, dissolved in 0.5 ml of sodium citrate (0.05 M), pH 4.5 (Sigma-Aldrich, St. Louis, MO, USA) to induce a diabetes-like condition.

One week after STZ injection (considering the first day after the second week as the zero day), whole blood samples were retrieved from the tail vein of the fasting rats, and their glucose levels were checked using Code Free blood glucose meter (Roche Diagnostics, Basel, Switzerland). Any Rats with glucose levels over 15 mmol/L (270 mg/dL) were included in the diabetic group and then randomly assigned into two subgroups: the SeNPs-treated diabetic (D-SeNPs) group and the control diabetic group (without any treatment). The D-SeNPs and the control SeNPs groups received SeNPs orally at a dose of 0.5 ml of SeNPs with concentration of 1 mg/ml dist H_2_O by oral administration for seven consecutive days (Al-Quraishy et al., 2015).

The rats were left for 24 h after taking the last dose, and then they were euthanized under mild ether anesthesia. They were sacrificed via fast decapitation; blood samples were collected in heparinized tube and were left for half an hour before being centrifuged at 5000 rpm for 15 min at 4 °C to separate plasma which was stored at -20 °C to be used for the various biochemical tests. Pancreas was dissected out then cut into pieces for further different studies. The first part was immediately fixed in a 10% phosphate buffered formaldehyde solution for histological and immunohistochemical analysis. The second part of pancreas was weighed and homogenized very well immediately to give 10% (w/v) homogenate in ice-cold medium containing 50 mM Tris–HCl, pH 7.4. The pancreas homogenates were then centrifuged at 5000 rpm for 10 min at 4 °C. The supernatant was stored in -70 °C until used for the various biochemical applications. Additional pancreatic tissues were used for further RNA extraction.

### 2.4 Biochemical investigations

#### 2.4.1 Blood glucose assay

Plasma glucose levels identically were measured via the glucose oxidase method according to Trinder (1969) using a glucose assay kit (Spectrum-Diagnostics, Cairo, Egypt).

#### 2.4.2 Plasma insulin determination

Plasma insulin levels were determined via the ELISA technique by using rat-specific kits purchased from BioVendor (Gunma, Japan) following to the protocol provided within the kit.

#### 2.4.3 Oxidative stress markers

Lipid peroxidation (LPO) was evaluated by using the reaction of thiobarbituric acid in homogenates (Ohkawa et al., 1979). Briefly, 100 mg of the homogenate was mixed with 100 μl of sodium thioglycolate (1%), 100 μl of 100% trichloroacetic acid (TCA), and 250 μl of 1 N HCl. After that, the mixture was incubated for 20 min at 100 °C then centrifuged at 4000 rpm for 10 min. The absorbance of the formed color was recorded at 532 nm. Furthermore, the nitrite/nitrate (nitric oxide (NO)) and glutathione (GSH) levels were detected by the methods of Green et al. (1982) and Ellman (1959), correspondingly. In NO assay, the evolution of the NO was carried out on the optimized acid reduction process in an acidic medium and in the presence of nitrite. Through this reaction, the nitrous acid diazotized sulfanilamide with N-(1–naphthyl) ethylenediamine, and the resultant bright reddish purple azo dye can be determined at wavelength 540 nm, whereas GSH was measured by the reduction of the 5,5′-dithiobis (2-nitrobenzoic acid) (Ellman's reagent) with GSH to form a yellow colored compound. However, the amount of reduced chromogen is directly proportional to the GSH concentration, and its absorbance can be determined at 405 nm. According to Paglia and Valentine (1967), the Glutathione peroxidase (GPx) was also evaluated. The assay is considered as an indirect measure of the potential activity of GPx. The Oxidized GSH, produced upon the reduction of organic peroxide by the GPx, is recycled to its reduced state via the enzyme glutathione reductase. Furthermore, the oxidation process of NADPH to NADP^+^ was accompanied by a decrease in the absorbance at 340 nm.

### 2.5 Histopathological examination

This examination were performed by taking a biopsy from the pancreas then was visualized under the light microscope. Firstly, it was washed with saline and then was fixed in neutral formalin (10%). After that, it was embedded in paraffin, sectioned at 5 μm and H&E staining was carried out.

### 2.6 Immunohistochemical analyses of insulin

Immunohistochemical staining for the insulin was carried out by using the avidin-biotin complex (ABC) method via a Rat/Mouse Insulin kit (Promega Co.). After de-waxing and rehydrating, the pancreatic samples taken were incubated in 0.03% hydrogen peroxide and then were washed and incubated with 5% normal goat serum. Subsequently, the samples were incubated with rabbit anti-insulin antibody that was previously diluted in phosphate-buffered saline (PBS). The samples then were washed three times in solution of 0.1 mol/L PBS afterwards, were incubated with biotinylated goat anti-rabbit antibody diluted in PBS. Then, they were washed again in 0.1 mol/L PBS followed by an incubation for 20 min at 37 °C using the avidin-biotin peroxidase complex. The sections were visualized with DAB/hydrogen peroxide and then they were rinsed, then stained with using hematoxylin, dehydrated, cleared, and well cover slipped. Samples were incubated with the same concentration of antibodies and under the same conditions. The staining intensities were classified as weak, very weak, medium, or strong.

### 2.7 Molecular assay (real time PCR)

RNeasy Plus Minikit (Qiagen, Valencia, CA) was utilized in order to isolate total RNA from pancreatic tissues to measure the expression level of the GPx gene. cDNA was synthesized by following the protocol provided with the iScript™ cDNA synthesis kit (Bio-Rad, CA). Real time PCR was carried out via Power SYBR® Green (Life Technologies, CA) and the Applied Biosystems, 7500 Instrument. The reference gene used was β-actin, and cDNA samples were run in triplicates. The thermal cycling program used was as follows: the heat was 95 °C for 10 min during the initial denaturation stage, 95 °C in the amplification phase for 10 s which was left to run 40–45 cycles, 66 °C for 10 s in the annealing phase, and finally 72 °C for 20 s in the extension phase. The single fluorescence was captured for each capillary to perform signal detection. The cycle threshold (CT) was measured for both target genes (β-actin and GPx gene). Subsequently, the relative quantization (RQ) for each sample was calculated using a specific formulas to normalize the expression towards the housekeeping gene also to be able to compare it with the control. At first, the Δ CT was measured for each sample, in where ^Δ^CT = CT related to the target gene – CT of the housekeeping gene. Secondly, the ^ΔΔ^CT was evaluated for each sample in which ^ΔΔ^CT = ^Δ^CT related to the experimental sample and – ^Δ^CT is for the control. Finally, the RQ was calculated where RQ = 2^-^^ΔΔCT^.

The PCR primers for GPx gene were designed by using the NCBI, Primer-Blast program were fabricated using Euro fins MWG Operon (Huntsville, AL). Their sequence was as follows:

GPx (S): 5′-CGGTTTCCCGTGCAATCAGT-3'.

GPx (AS): 5′-ACACCGGGGACCAAATGATG-3'.

β-Actin (S): 5′-GGCATCCTGACCCTGAAGTA-3'.

β-Actin (AS): GGGGTGTTGAAGGTCTCAAA-3'.

### 2.8 Statistical analysis

The one-way analysis of variance (ANOVA) was utilized with data for multiple variable comparisons. Duncan's test was performed via the statistical package program (SPSS version 17.0), while the resulted figures were drawn with Prism program (version 6.1) for evaluation of significance comparison of the difference between the groups tested. The mean ± standard error of the mean (SEM) was calculated for all the results. The p values were two-tailed and p < 0.05 was considered as a significant.

## 3 Results

### 3.1 Characterization of the prepared SeNPs

**Figure 1:**
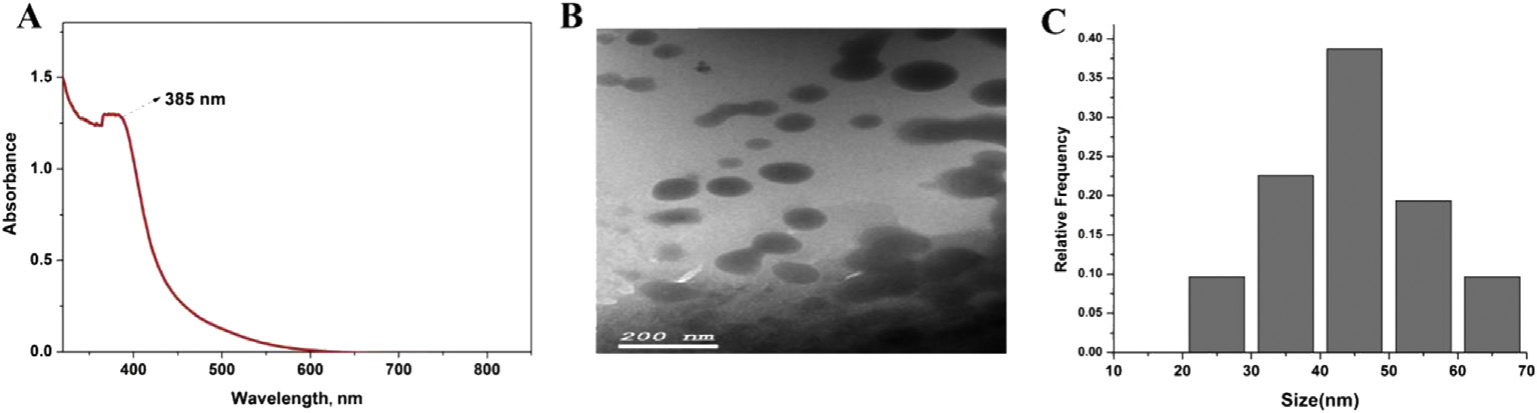
SeNPs characterizations. A: Absorption spectrum; B: TEM image with scale bar = 200 nm; C: Histogram shows the frequency of NPs with different sizes.

The formation of SeNPs was confirmed by the appearance of a peak in the UV region upon scanning on spectrophotometer. This band was observed at 385 nm as seen in Figure 1A. The morphology of formed NPs such as particle shape and size was investigated via TEM analysis. SeNPs were formed and showed a semi-spherical shape, with a size around 45 nm as shown in Figure 1B. This was evident in the histogram calculated for the images taken: while there were SeNPs different in size, particles that are sized 40–50 nm were the most abundant as shown in Figure 1C.

### 3.2 Biochemical investigations

#### 3.2.1 Glucose and insulin results

A significant decrease in the weight gain of the STZ-intected rats was observed during the experiment after the induction of diabetes. Furthermore, the food consumption significantly declined. Diabetic rats also had polyuria (data not shown). However, these symptoms were significantly attenuated upon the SeNPs treatment.

**Figure 2:**
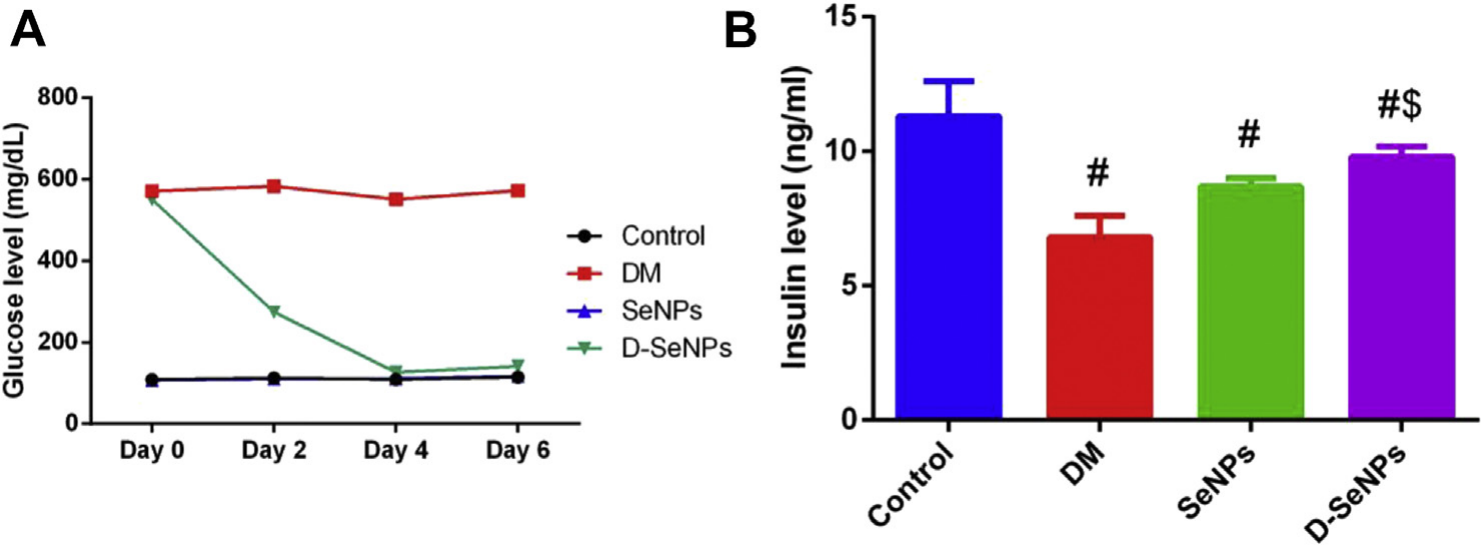
A: SeNPs effect on the plasma glucose levels over six days of SeNPs treatment; B: Effect of SeNPs on plasma insulin level of the control and tested experimental rats. The values are mean ± SEM (n = 6); ^#^p < 0.05, the significant change with versus the control group; ^$^p < 0.05, the significant change with versus the DM group. DM: Diabetes mellitus; SeNPs: SeNPs only; D-SeNPs: SeNPs-treated diabetic group.

In the current study, every two days after seven days of diabetes induction, plasma glucose levels were measured for all groups. The glucose level did not change during the experimental period in both the control and SeNPs groups. However, a noteworthy increase in glucose level was noticed in the diabetes groups confirming that they became diabetic. As shown in Figure 2A, after administering SeNPs to diabetic rats, a marked reduction in glucose level was noted in comparison with the diabetic untreated rats. Simultaneously, insulin levels drastically declined in the diabetes groups compared to the control rats (p < 0.05), but treating with SeNPs restored the level of plasma insulin in the D-SeNPs group as shown in Figure 2B.

#### 3.2.2 Oxidative stress markers results

**Figure 3:**
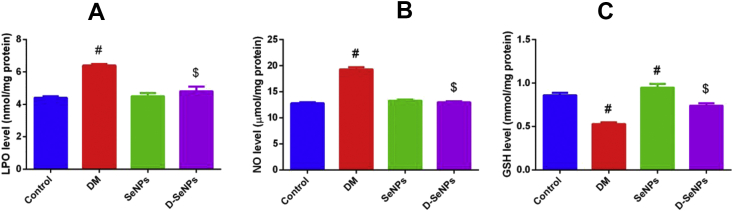
SeNPs effect on oxidative stress indicators of the control and tested experimental rats. A: lipid peroxidation; B: nitric oxide; C: glutathione. The values are mean ± SEM (n = 6); ^#^p < 0.05, a significant change with versus the control group; ^$^p < 0.05, a significant change with versus the DM group. DM: Diabetes mellitus; SeNPs: SeNPs only; D-SeNPs: SeNPs-treated diabetic group; LPO: lipid peroxidation; NO: nitric oxide; GSH: glutathione.

**Figure 4:**
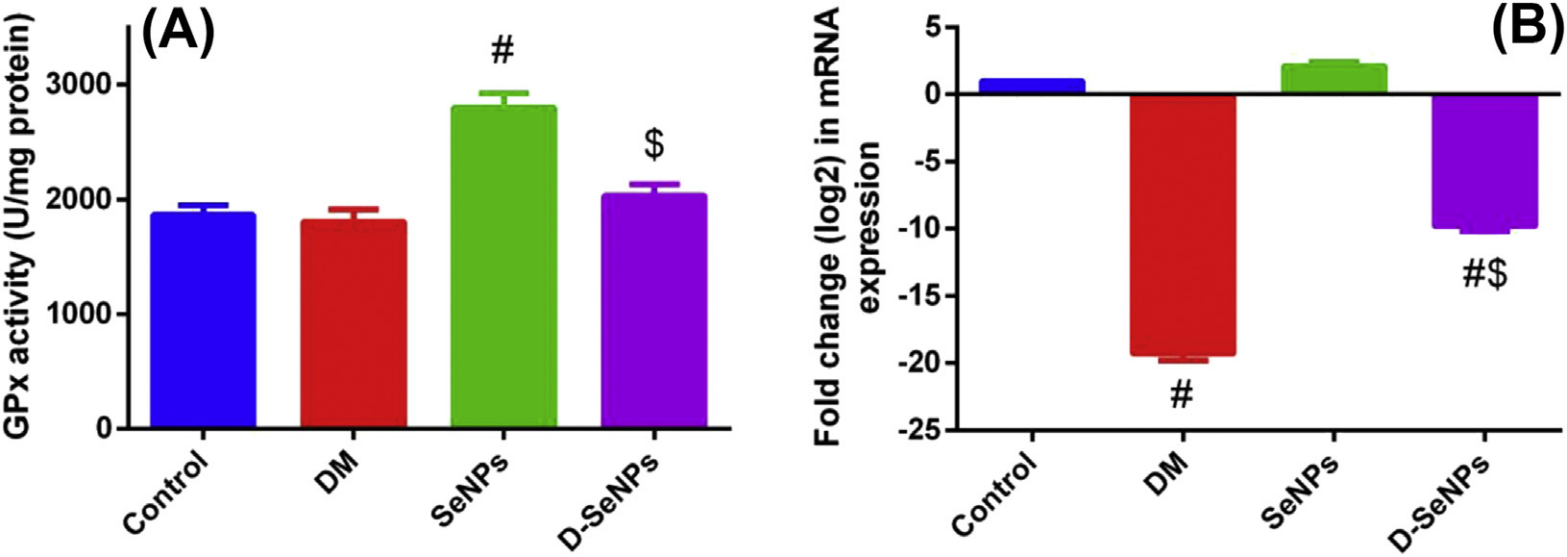
SeNPs effect on (A) GPx activity and (B) mRNA expression level of control and experimental groups. For activity: the values are mean ± SEM (n = 6); for expression: the values (mean of three assays ±SEM) were exactly normalized to β-actin RNA level and are shown as fold induction relative to the mRNA level in the control. ^#^p < 0.05, significant change with versus the control group; ^$^p < 0.05, significant change with versus the DM group. DM: Diabetes mellitus; SeNPs: SeNPs only; D-SeNPs: SeNPs-treated diabetic group; GPx: glutathione peroxidase.

In this study, the NO, LPO and GSH levels were assessed for the four groups in pancreatic homogenates as shown in Figure 3A. LPO in the pancreatic tissues significantly lowered (p < 0.05) in D-SeNPs group in comparison with the diabetic untreated rats which had the highest LPO level. Nitric oxide decreased drastically (p < 0.05) in D-SeNPs rats compared to the diabetic untreated ones that possess the highest NO level. Moreover, the level of pancreatic NO was slightly raised in the SeNPs group compared to the control rats (Figure 3B). GSH content in the pancreatic tissues was markedly elevated in the Se control rats, while it reduced in the diabetic untreated rats as compared to the control rats (p < 0.05). GSH levels in the D-SeNPs group significantly elevated compared to the diabetic untreated rats (p < 0.05) (Figure 3C). In order to prove the impact of SeNPs on the antioxidant activity, GPx activity was evaluated. As shown in Figure 4A, the highest level of GPx activity was found in the SeNPs control group. The activity of GPx stimulated in the D-SeNPs group when compared to the diabetic untreated rats.

Consistent with the biochemical findings, the expression of GPx gene was quantified by using real time PCR via the comparative CT method (^ΔΔ^CT method) as shown in Figure 4B. The RQ of the gene in the samples was compared to the control sample. Results revealed that the expression of the GPx gene in the Se control group was one-fold higher than the control sample, while the expression in the diabetic untreated rats was much lower. The D-SeNPs group showed low expression of the gene in comparison with the control, but it is still higher than the diabetic control which indicated a rise of expression of the GPx gene after SeNPs treatment.

### 3.3 Histopathology findings

**Figure 5:**
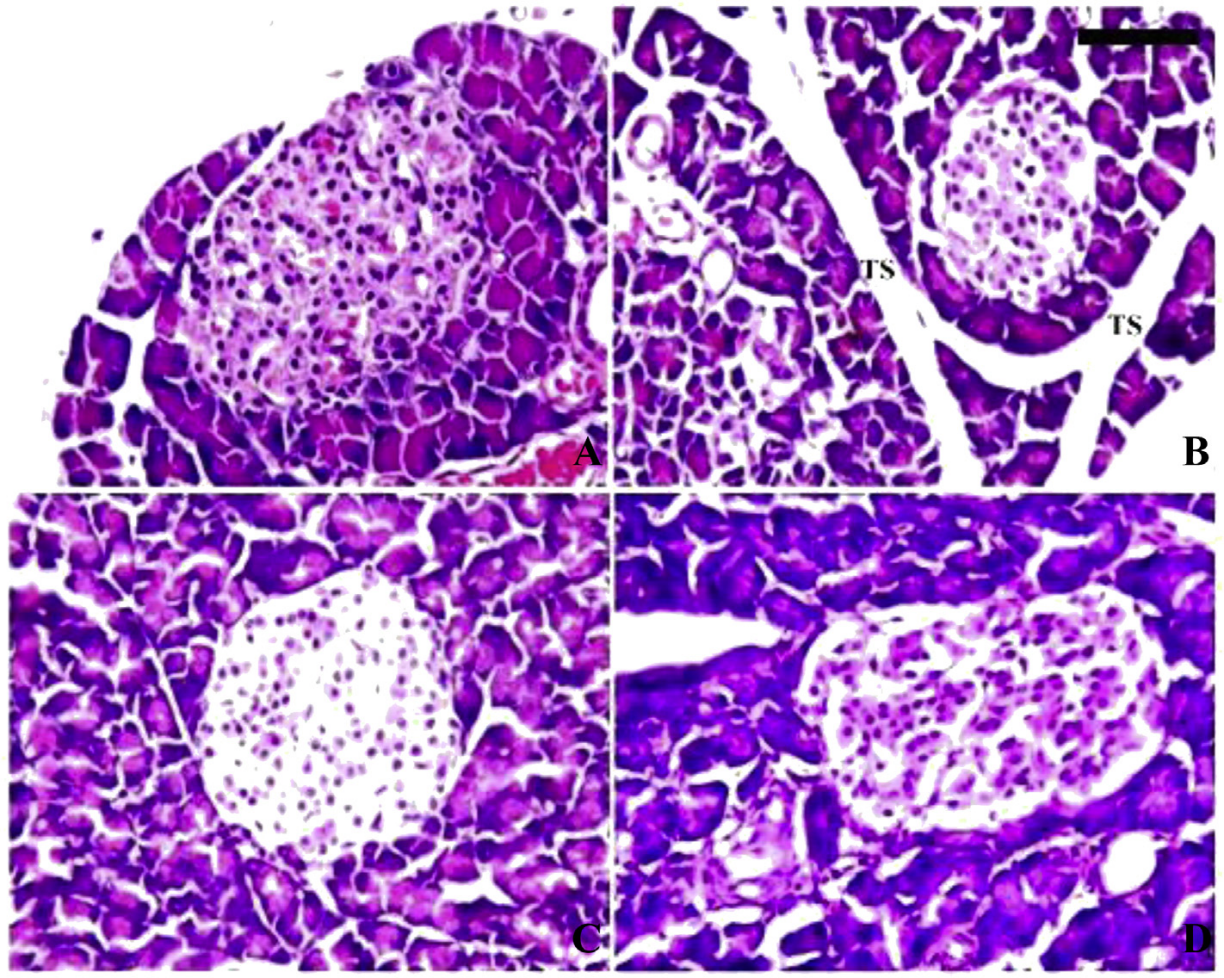
Histology of the pancreas. A: Control group, revealing normal texture pattern with contact islets of Langerhans; B: STZ group, displaying degenerative shrunken islets of Langerhans and necrotic changes; C: SeNPs-treated group, showing normal texture pattern with contact islets of Langerhans. D: D-SeNPs-treated group, SeNPs protected the majority of cells in the islet of Langerhans (400×; scale bar = 100 μm).

The pancreas of the control group and Se control groups were showed a normal texture pattern with a contact islets of Langerhans and normal thickness septa (TS) (Figure 5a and c, respectively). The TS of the pancreas in the diabetic group was wider than the remaining groups. The islets of Langerhans showed signs of atrophy and shrinking. Infiltrated lymphocytes were seen which indicates inflammation of these cells (Figure 5b). As shown in Figure 5d, the islets of Langerhans were regenerated and mainly restored their normal shape in the diabetic rats treated with SeNPs. Furthermore, the measured diameters of the islets of Langerhans area were increased in D-SeNPs-treated rats in comparison with the diabetic untreated rats.

### 3.4 Immunohistochemistry results

**Figure 6:**
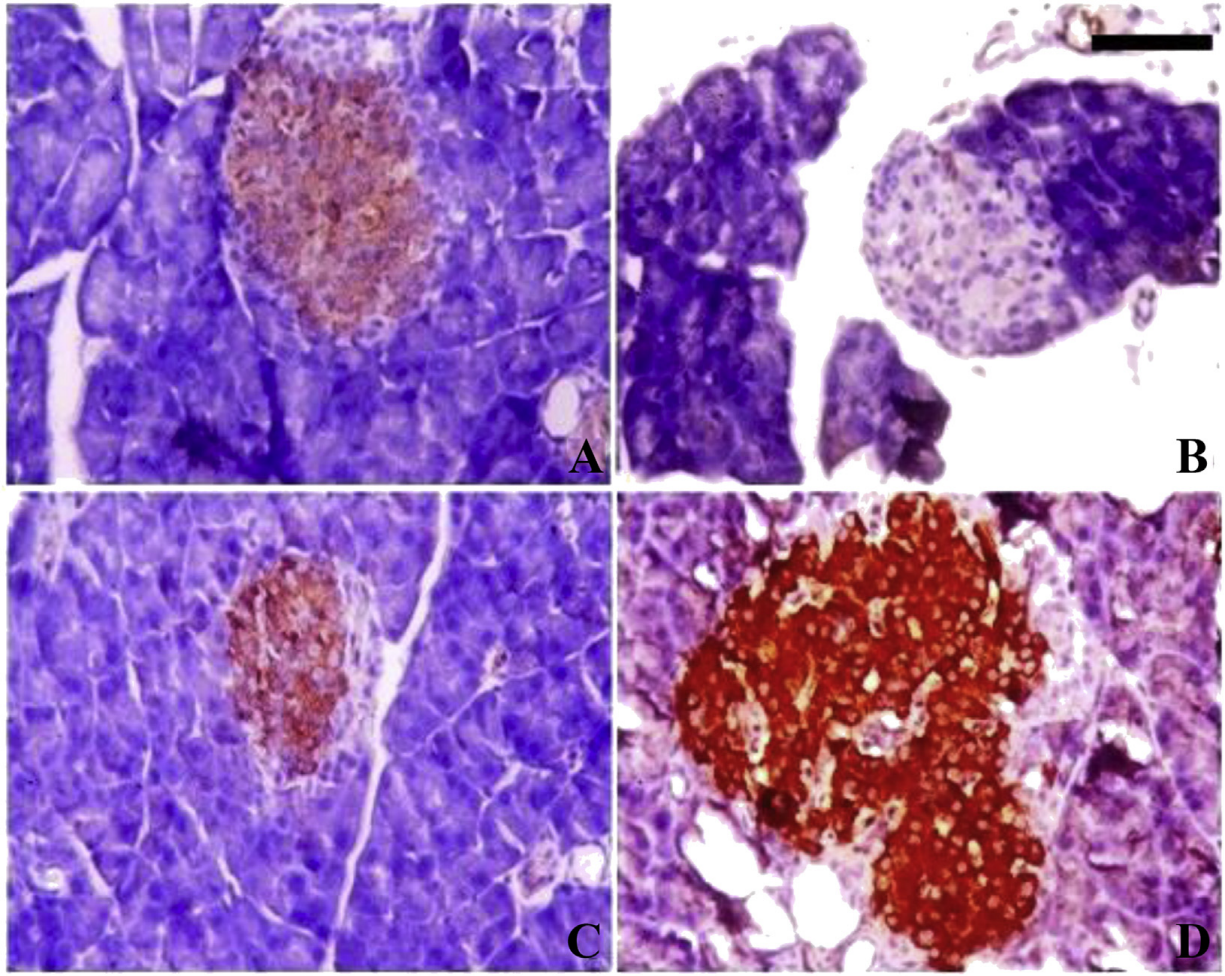
Pancreas sections stained with insulin antibody. A: Control group, β-cells in the islet of Langerhans are strongly stained with the anti-insulin antibody; B: STZ group, weak insulin-immunoreactivity is exhibited in a few β-cells in the islet of Langerhans in diabetic rats; C: SeNPs group, showing β-cells in the islet of Langerhans that are weakly stained with the anti-insulin antibody. D: D-SeNPs-treated group, SeNPs has protected the majority of β-cells in the islet of Langerhans and strong staining with the anti-insulin antibody is shown (400×; scale bar = 100 μm).

Immunohistochemistry was performed to examine insulin synthesis on the pancreatic islets. In the normal control rats, the majority of cells within islets were positively stained to insulin with moderate intensity as seen in Figure 6a. The immunostaining potential of insulin was decreased obviously in the pancreatic islets of Langerhans in diabetic rats (Figure 6b). This immunostaining activity for insulin was noticeably increased upon treating with SeNPs (Figure 6c). Interestingly, the section from D-SeNPs-treated group showed strong immunostaining activity for insulin when compared to the control and diabetes untreated rats as shown in Figure 6d.

## 4 Discussion

Diabetes is considered to be a cluster of several metabolic disorders (Rojas and Gomes, 2013). Chronic increased blood glucose levels cause irreversible changes to different tissues. High blood glucose levels increase the oxidative pressure in the body because advanced glycation end-products (AGE) are produced which stimulates the reactive oxygen species (ROS) production. Complications of diabetes appear primarily due to oxidative pressure (Singh et al., 2014).

Using antioxidants as therapeutic agents for diabetes has been examined extensively. According to Tabatabaei-Malazy et al. (2013), natural antioxidants such as quercetin and curcumin protected β-cells from apoptosis and preserved their function, and they decreased oxidative stress. Similarly, Se has been evaluated as an anti-diabetic agent for its natural antioxidant activity; however, it has been reported that it may be toxic if administered with high concentrations (Ramamurthy et al., 2013). Contrarily, SeNPs are biocompatible, and they have a stronger effect due to their tiny size and high surface area (AlBasher et al., 2019).

SeNPs were used in this research to evaluate a new therapeutic approach for type 2 diabetes. The synthesis of SeNPs was done by following a successful method, where glucose was used as a reducing agent and PVP as a stabilizing agent (Ingole et al., 2010). This synthesis method is unique and it is recommended because it is considered green, eco-friendly and biocompatible. PVP was used as a capping agent to increase the stabilization of the particles and prevent them from aggregation and because it is an organic biocompatible polymer (Stroyuk et al., 2008).

In this research, STZ-induced diabetic rats were treated with the SeNPs for a week causing noticeable decrease in glucose levels. These findings were comparable to the findings of Vural et al. (2017), who treated diabetic rats with sodium selenite and glucose levels decreased and remained low for seven weeks. Se is an insulin mimetic, which explains its ability to reduce the levels of glucose (Abdulmalek and Balbaa, 2019). According to Hwang et al. (2007), Se increases the transport of glucose because it has insulin-like activity both *in vitro* and *in vivo*. Ezaki (1990) reported that selenate enhanced the transportation and uptake of glucose in adipocytes of rats by translocating glucose transporters, such as GLUT-1 and GLUT-2, to the surface of many membranes. Furthermore, the glucose lowering effect of Se might be supported by other mechanisms, such as an acceleration of kidney glucose excretion in rats or stimulation of adipogenesis in adipocytes via stimulating serine/threonine kinases, including the p70 S6 kinase (Al-Quraishy et al., 2015).

The insulin levels in two groups, the Se control and the SeNPs-treated diabetic rats, were affected after treatment. As shown in Figure 2B, the level of plasma insulin was drastically reduced in the SeNPs control group as compared to the control group. These results coincide with the study of Wang et al. (2017), who concluded that the Se control group did not need to secrete insulin due to the insulin-like properties of Se and also due to the ability of Se to reduce the insulin resistance. However, the moderate immunostaining activity of insulin in the Se group suggested that the synthesized insulin was un-secreted and stored in the β-cells. However, the insulin level of the D-SeNPs group increased significantly in comparison with the diabetic control group. This suggests that the SeNPs has increased the level of insulin secretion and regenerated the pancreatic cells. Tong and Wang (1998) concluded in their study that the ability of the pancreatic islets to secrete insulin is increased when rats were fed with a Se-supplemented diet. According to Campbell et al. (2008), the raise in insulin production was attributed to the ability of Se to enhance the function of β-cells in rats by upregulating the expression of insulin promoter factor 1 (Ipf1) gene which is one of the most important transcription factors in controlling the production of insulin in β-cells.

In the present study, LPO level was increased drastically in diabetic rats. Peroxides-mediated tissue injure has been exhibited in the development of both types 1 and 2 diabetes (T1D and T2D). Hyperglycemia and STZ-induced diabetes in animals increased the formation of free radicals that react with polyunsaturated fatty acids in the cell membranes, leading to LPO which increases production of free radicals (Uyoyo Ukperoro et al., 2010). However, in this investigation, results suggest that SeNPs had significant protective effects against diabetes-induced oxidative stress. Administration of SeNPs decreased pancreatic LPO in the diabetic group. These results coincide with the work of Vural et al. (2017), who reported that Se treatment could significantly reduce the elevated LPO in diabetic rats. There have been many investigations that have proven that antioxidant administration decreases augmented lipid peroxide in diabetic rats (El-Alfy et al., 2005) and hyperlipidemia and vascular damage in ApoE-knockout mice (Guo et al., 2019). Furthermore, Bai et al. (2020) recently reported that SeNPs have a potent antioxidant property.

In the current study, significant elevated levels of NO were exhibited in the pancreas of the untreated diabetic group. Inflammation and lesion of pancreatic islets and the impairment of β-cells have been linked to the elevated presence of NO (Cernea and Dobreanu, 2013). NO is produced by inducible nitric oxide synthase (iNOS) in inflammatory macrophages (El-Mahmoudy et al., 2005), and in the inflamed pancreatic β-cells themselves (Corbett et al., 1993). Increased production of the toxic inflammatory molecule, NO, may lead to β-cells death by causing breakages in the DNA, modifying proteins, and inhibiting metabolism in the mitochondria (Bae et al., 2013; Montgomery and Turner, 2015; Song et al., 2010).

GHS is a tripeptide that is usually examined to indicate the level of oxidative stress. Usually, GSH regulates the redox status of cells in which it decreases oxidative stress. The function of GSH is to bind to oxidants and to dispose them. It protects cells against ROS. In this research, GSH levels were drastically depleted (p < 0.05) in the pancreatic tissues of the diabetic rats. SeNPs treatment significantly restored GSH contents in the diabetic rats as shown in Figure 3C. Asemi et al. (2015), supplemented gestational diabetic patients with Se in a randomized control trial for six weeks and their results also showed an increase in GSH. This increase is correlated with the antioxidant effect of Se which allows Se to bind to ROS, thus preventing it from converting GSH to oxidizing GSH (GSSG) (Abdelfattah et al., 2019).

SeNPs decreased the level of ROS significantly; this could be due to its incorporation in one of the most important antioxidant enzymes, GPx. GPx activity indicates the level of Se and selenoprotein in a body (Dkhil et al., 2016). As shown in Figure 4B, SeNPs administration to diabetic rats significantly enhanced activity and mRNA expression of GPx and restored the levels to normal in comparison with the diabetic untreated rats. This illustrates the potential of using SeNPs in managing type 2 diabetes because GPx is of crucial importance, where it scavenges free radicals like NO and decreases oxidative stress (Kumar et al., 2014). Furthermore, it also increased the levels of GPx in the Se control group in comparison with the normal control. Campbell et al. (2008) reported increase in GPx activity in rats treated with selenite. In this study, the Se control group showed higher Se levels and this could be because the oxidative pressure is more in the diabetic rats and thus the GPx will be more utilized in the diabetic rats than in the control ones.

In the histological evaluation of the pancreatic tissues, the diabetic control showed damage in the β islets as shown in Figure 5b. According to Deeds et al. (2011), STZ causes eventual β-cell death by causing alkylation of the DNA. On the other hand, the SeNPs significantly restored the normal shape of the pancreatic islets as shown in Figure 5d. According to Tong and Wang (1998), Se restored the morphological shape of islet β-cells in Keshan's disease and protected the pancreatic cells from additional deterioration. Furthermore, in a study by Chang et al. (2009), with other micronutrients, Se elevated insulin levels on the transcriptional and translational levels in STZ-induced diabetic mice, and it demonstrated protective capabilities by restoring the morphological shape of damaged β-cells. Furthermore, Wang et al. (2019) reported that SeNPs functionalized with a polysaccharide isolated from *Rosa roxburghii* fruit limited the dysfunction of β-cells by inhibiting apoptosis and oxidative stress.

## 5 Conclusion

In this research, SeNPs were successfully synthesized via a green method using glucose and a biodegradable polymer (PVP); they were characterized then utilized to reduce the hyperglycemia by decreasing the plasma glucose levels. It also increased the levels of insulin both in the plasma and the pancreas, and it restored the damaged pancreatic tissues of the diabetic rats. SeNPs administration decreased oxidative stress and increased GPx enzyme both on the transcriptional and the cellular levels. With more extensive research, SeNPs could be used in the future as an agent that could manage diabetes.

## Declarations

### Author contribution statement

Ola M. El-Borady, Mohamed S. Othman and Ahmed E. Abdel Moneim: Conceived and designed the experiments; Performed the experiments; Analyzed and interpreted the data; Contributed reagents, materials, analysis tools or data; Wrote the paper.

Heba H. Atallah: Performed the experiments; Contributed reagents, materials, analysis tools or data; Wrote the paper.

### Funding statement

This research did not receive any specific grant from funding agencies in the public, commercial, or not-for-profit sectors.

### Competing interest statement

The authors declare no conflict of interest.

### Additional information

No additional information is available for this paper.
